# Drug-induced immune-mediated thrombocytopenia secondary to sunitinib in a patient with metastatic renal cell carcinoma: a case report

**DOI:** 10.1186/1752-1947-7-54

**Published:** 2013-02-26

**Authors:** Zia Ansari, Mathew K George

**Affiliations:** 1 Department of Medical Oncology, Tamworth Rural Referral Hospital, Locked Mail Bag 9783, 2348, Tamworth, NEMSC NSW, Australia

**Keywords:** Metastatic renal cell carcinoma, Sunitinib, Thrombocytopenia

## Abstract

**Introduction:**

Sunitinib is an oral multi-targeted tyrosine kinase inhibitor approved for first line treatment for metastatic renal cell carcinoma and imatinib-resistant metastatic gastrointestinal stromal tumors. Sunitinib administration can cause myelosuppression resulting in neutropenia and thrombocytopenia. Here we present the case of a patient with metastatic renal cell carcinoma who developed sunitinib-induced immune-mediated thrombocytopenia and who was treated with withdrawal of sunitinib and administration of intravenous immunoglobulin and steroids.

**Case presentation:**

This case report describes a 70-year-old Aboriginal Australian with a diagnosis of metastatic renal cell carcinoma. Three weeks after the initiation of sunitinib he developed epistaxis and was admitted with thrombocytopenia (platelets 7 × 109/L) which was found to be refractory to platelet transfusion. Sunitinib was stopped and he was treated with intravenous immunoglobulin and steroids. His platelet count rapidly improved and returned to baseline in three weeks. Only two cases of sunitinib-induced immune-mediated thrombocytopenia have been described in the literature.

**Conclusion:**

Clinicians should have a high index of suspicion for the potential of immune-mediated thrombocytopenia after the initiation of multi-targeted tyrosine kinase inhibitors such as sunitinib. This is a diagnosis of exclusion and can be safely treated by drug withdrawal.

## Introduction

Sunitinib (Sutent®) is an oral multi-targeted tyrosine kinase inhibitor. Sunitinib inhibits members of the split-kinase domain family of receptor tyrosine kinases (RTKs) including the vascular endothelial growth factor receptors (VEGFRs) types 1 and 2 (FLT1 and FLK1 or KDR); platelet-derived growth factor receptors alpha and beta (PDGFR-alpha and PDGFR-beta); the stem cell factor receptor c-KIT; and the *FLT3* and *RET* kinases. Inhibition of these RTKs results in a reduction in tumor growth, progression, metastases and angiogenesis [1]. Clinically sunitinib is approved for the first line treatment of metastatic renal cell carcinoma (mRCC) and imatinib-resistant metastatic gastrointestinal stromal tumors. Reported toxicities of sunitinib include fatigue, hypertension, diarrhea, vomiting, skin toxicity (hand and foot syndrome), neutropenia and thrombocytopenia [2]. Here we present the case report of a patient with mRCC who developed sunitinib-induced immune-mediated thrombocytopenia and recovered after the withdrawal of sunitinib and immunoglobulin and steroid support.

## Case presentation

The patient is a 70-year-old Aboriginal Australian with a history of a left nephrectomy in 2005 for clear cell renal cell carcinoma as well as multiple co-morbidities including chronic obstructive airway disease, ischemic heart disease with coronary artery bypass graft, aortic valve replacement on warfarin and gastroesophageal reflux disease. His medications included fluticasone and salmeterol inhaler (250 and 50mcg respectively) two puffs twice a day, furosemide 20mg in the morning, atorvastatin 40mg at night, ranitidine 300mg in the morning, and paracetamol 1g daily. Investigation for shortness of breath revealed multiple metastases in both lungs, the biopsy of which confirmed mRCC. There was no previous history of autoimmune disease, hematological disorder, liver disease, human immunodeficiency virus, or hepatitis B or hepatitis C infection. His baseline full blood count revealed: hemoglobin 131g/L, white blood cell count 6.4 × 10^9^/L and platelets 294 × 10^9^/L. He was commenced on sunitinib 50mg/day. The patient did not take any new medications, herbal or over the counter drugs since his commencement of sunitinib. There was no evidence of liver metastases.

A routine full blood count two weeks post-treatment showed a decline in his platelets to 129 × 10^9^/L, however, his hemoglobin was 161g/L and white blood cell count was 4.9 × 10^9^/L. In the third week he developed epistaxis and was admitted to hospital. His platelets dropped to 7 × 10^9^/L and his international normalized ratio (INR) was 2.4. This was reversed with an intravenous vitamin K injection. His sunitinib and warfarin were stopped. The epistaxis stabilized with nasal packing and he received a platelet transfusion. His thrombocytopenia did not respond and his platelet count dropped further to 1 × 10^9^/L.

On clinical examination there was evidence of oropharyngeal petechiae, epistaxis and peripheral ecchymoses. There was no fever, lymphadenopathy, hepatosplenomegaly or neurological signs. Laboratory investigations included normal renal function tests, electrolytes and stable liver function tests. Coagulation screening showed his INR had reversed to 1.1 after intravenous vitamin K, prothrombin time 12 seconds (11 to 15), activated partial thromboplastin time 24 seconds (23 to 38) and fibrinogen 3.7g/L (2.0 to 4.0). Peripheral blood film showed thrombocytopenia and no evidence of schistocytosis, spherocytosis or dysplasia. There was no evidence of hemolysis.

Disseminated intravascular coagulation and thrombotic microangiopathy were ruled out as possible causes of sunitinib-mediated thrombocytopenia by the results of the above investigations. Platelet-bound immunoglobulin and a bone marrow aspirate were not performed when discussed with a hematologist, and the diagnosis of exclusion, sunitinib-induced immune-mediated thrombocytopenia, was made. The patient was treated with intravenous immunoglobulin 27.5g (0.4g/kg) once daily for five days with prednisolone 50mg once a day. His platelet count rapidly improved to 103 × 10^9^/L and returned to a baseline of 259 × 10^9^/L after three weeks.

Normalization of this patient’s platelet count following withholding of sunitinib is consistent with the diagnosis of immune-mediated thrombocytopenia secondary to sunitinib.

## Discussion

Drug-induced immune-mediated thrombocytopenia is thought to be a result of antibody production in the presence of a sensitizing medication, with the antibodies targeting epitopes on the platelet surface, subsequently leading to the clearance of the antibody-coated platelets by the mononuclear phagocytic system. It takes five to seven days of exposure to produce sensitization in a patient given the drug for the first time. Although drug-induced thrombocytopenia is uncommon, it can have devastating, and even fatal, consequences that can usually be prevented simply by discontinuing the causative drug. It is therefore important that clinicians have a general understanding of this condition and the drugs that can cause it. Chemotherapeutic and immunosuppressive agents typically cause thrombocytopenia by suppressing hematopoiesis; they can also cause immune-mediated thrombocytopenia [3]. Drug-induced thrombocytopenia should be suspected, therefore, in patients treated with such drugs if there is an acute drop in the platelet level after exposure.

Thrombotic microangiopathy is another cause of sunitinib-mediated thrombocytopenia, reported by Kapiteijn *et al*. [4]. Sunitinib is a tyrosine kinase inhibitor that targets VEGFR-2 and PDGFR-beta, which are both the major expression subtypes of VEGFR and PDGFR in capillary vasculature. It is hypothesized that sunitinib acts via direct anti-VEGFR and anti-PDGFR effects that result in damage of the capillary endothelium [4].

Sunitinib has been associated with other autoimmune disorders such as hypothyroidism; therefore, the possibility of an immunologic phenomenon to account for sunitinib-induced immune-mediated thrombocytopenia is reasonable [5,6]. However, the exact mechanism by which sunitinib induces an immune-mediated thrombocytopenia is unknown. In a phase III trial of metastatic renal cell cancer patients, Motzer *et al*. reported some grade of thrombocytopenia in 65 of 375 patients randomized to sunitinib treatment, with eight of the 65 (12%) having grade 3 thrombocytopenia [7].

Renal cell carcinoma is also associated with immune-mediated thrombocytopenia [8,9]. Therefore, we cannot rule out the possibility that the immune-mediated thrombocytopenia was related to renal cell carcinoma. However, in our case, sunitinib administration after the diagnosis of renal cell carcinoma resulting in thrombocytopenia resolving completely upon drug withdrawal is consistent with sunitinib-induced immune-mediated thrombocytopenia.

There have been two cases of sunitinib-induced immune-mediated thrombocytopenia reported to date. In both cases patients were treated with intravenous immunoglobulin 1g/kg over two days and intravenous tranexamic acid [10,11].

## Conclusion

In this case thrombocytopenia following the initiation of the multi-targeted tyrosine kinase inhibitors, sunitinib, was safely treated by drug withdrawal. When treating patients with these agents clinicians should have a high index of suspicion for the potential of immune-mediated thrombocytopenia which is a diagnosis of exclusion.

## Consent

Written informed consent was obtained from the patient for publication of this case report. A copy of the written consent is available for review by the Editor-in-Chief of this journal.

## Competing interests

The authors declare that they have no competing interests.

## Authors’ contributions

ZA and MG were both involved in the patient diagnosis and management. ZA conducted the literature review and carried out the review of the patient's medical record. MG participated in the preparation of the manuscript and revised the article for intellectual content details. Both authors read and approved the final manuscript.

## Authors’ information

ZA is an advanced trainee in Medical Oncology at Tamworth Rural Referral Hospital. MG is a Medical Oncologist at Tamworth Rural Referral Hospital.
